# Undiagnosed mirror syndrome with maternal hypoxemia onset during an emergency cesarean section: A case report

**DOI:** 10.1097/MD.0000000000040838

**Published:** 2024-12-13

**Authors:** Shino Ichikawa, Junichi Saito, Satoko Noguchi, Kurumi Saito, Kazuyoshi Hirota

**Affiliations:** a Department of Anesthesiology, Hirosaki University Graduate School of Medicine, Hirosaki, Japan; b Department of Perioperative Stress Management, Hirosaki, Japan; c Aomori Prefectural Central Hospital, Aomori, Japan.

**Keywords:** fetal hydrops, hypoxemia, maternal edema, mirror syndrome

## Abstract

**Rationale:**

Mirror syndrome is a rare pregnancy condition in which maternal edema is associated with fetal hydrops. Because of its rarity and overlapping symptoms, this condition is often misdiagnosed as another pregnancy complication.

**Patient concerns:**

A 28-year-old pregnant Japanese woman presented with sudden 7.5-kg weight gain, leg edema, and increased d-dimer level.

**Diagnoses:**

Ultrasound revealed polyhydramnios and fetal hydrops, and findings of maternal edema and blood test results were suggestive of Miller syndrome. Although, the patient was initially misdiagnosed due to a lack of information and the rarity of this disease.

**Interventions:**

An emergency cesarean section was performed under spinal anesthesia at 36 weeks and 2 days of- pregnancy. We could not diagnose mirror syndrome.

**Outcomes:**

The newborn’s Apgar scores were 2 and 5 at 1 and 5 minutes after delivery, respectively. The patient’s SpO_2_ suddenly decreased to 86% during cesarean section and persisted for 2 days. Chest computed tomography revealed pleural effusion and pulmonary edema. The pleural effusions and lung edema spontaneously resolved after the cesarean section.

**Lessons:**

This case reports on Miller syndrome with maternal hypoxemia onset during an emergency cesarean section and highlights the potential for better perioperative management and improvement in maternal mortality through prompt diagnosis and appropriate treatment shared not only among obstetricians and pediatricians but also among anesthesiologists.

## 1. Introduction

Mirror syndrome is a rare maternal pathology that “mirrors” fetal pathology and has a significant impact on fetal and maternal mortalities. Although the diagnostic criteria of mirror syndrome have not been clearly defined,^[1]^ the syndrome is characterized by 3 complications: fetal hydrops, placental edema, and maternal edema. Most cases of mirror syndrome are complicated by hypertension, and it is important to differentiate hypertension in mirror syndrome from hypertensive disorders during pregnancy.^[1,2]^ Generally, the causes of polyhydramnios and fetal hydrops include fetal (morphological and chromosomal abnormalities), maternal (impaired glucose tolerance), placental, and idiopathic factors.^[3]^ The relationship between maternal edema and fetal hydrops in patients with mirror syndrome has not been clarified.

The symptoms and laboratory findings of pregnant women with mirror syndrome resolve after intrauterine fetal death or delivery.^[1,2]^ In fetal hydrops, preterm delivery is recommended only in cases with specific obstetric indications, including the development of mirror syndrome.^[3]^ The prevalence of mirror syndrome may be higher than previously reported because of its undefined diagnostic criteria and low recognition of this syndrome. In existing reports, mirror syndrome was accompanied by signs of fetal hydrops, maternal edema, and hypertension.^[2,4]^ Early diagnosis and treatment based on shared knowledge of mirror syndrome among obstetricians, neonatologists, and anesthesiologists will contribute to a reduction in fetal and maternal mortality due to this condition. We report a case of sudden unexpected hypoxemia during cesarean section, which was later diagnosed as mirror syndrome based on fetal hydrops, maternal edema, and lung edema/pleural effusions.

Written informed consent was obtained from the patient for the publication of this case report, which adhered to the applicable CARE checklist.

## 2. Case presentation

**Figure 1.**
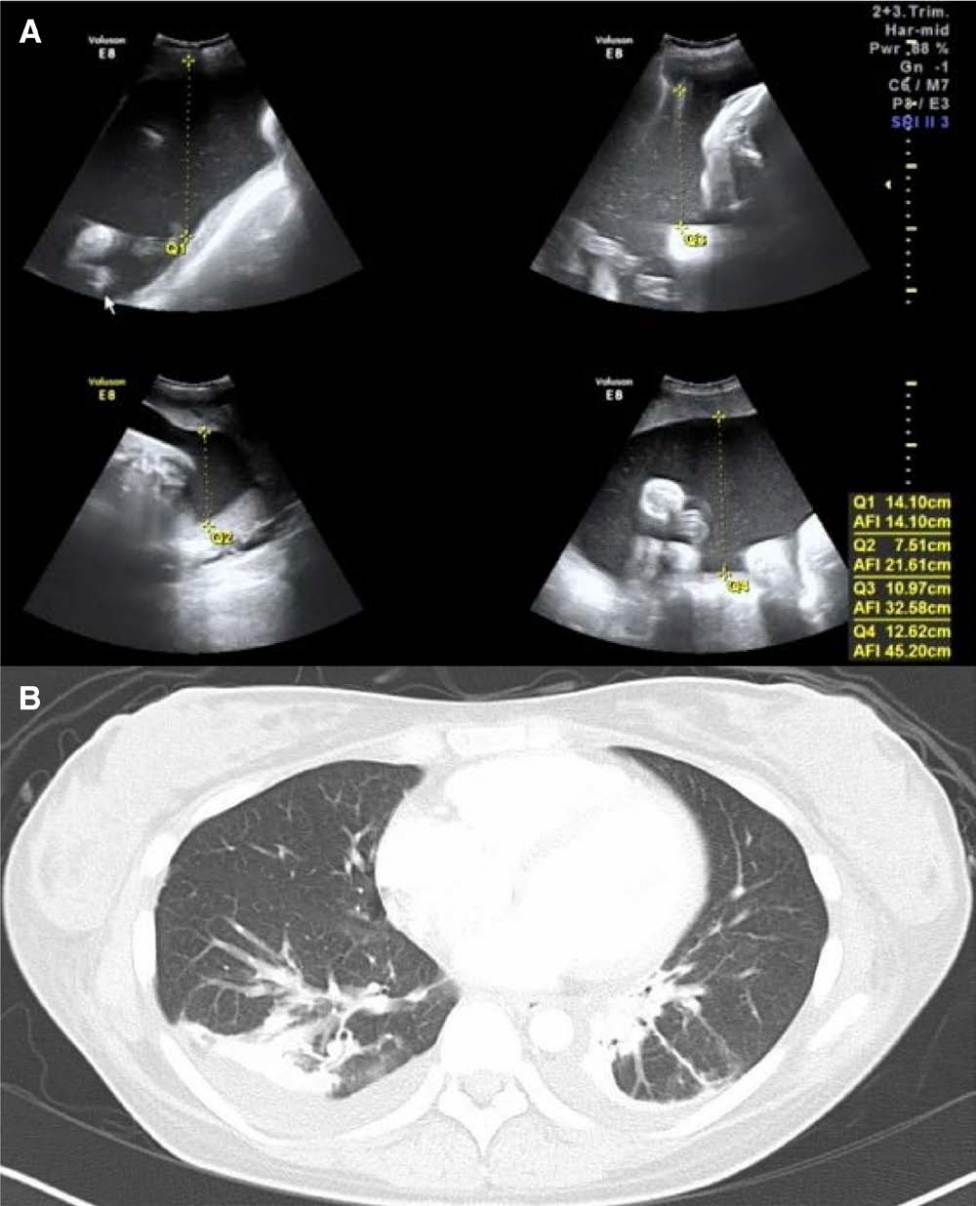
(A) Fetal ultrasound image at 36 weeks of gestation showing polyhydramnios. (B) Chest CT at 2 days after the delivery showed bilateral pleural effusions and mild pulmonary edema. CT = computed tomography.

**Figure 2.**
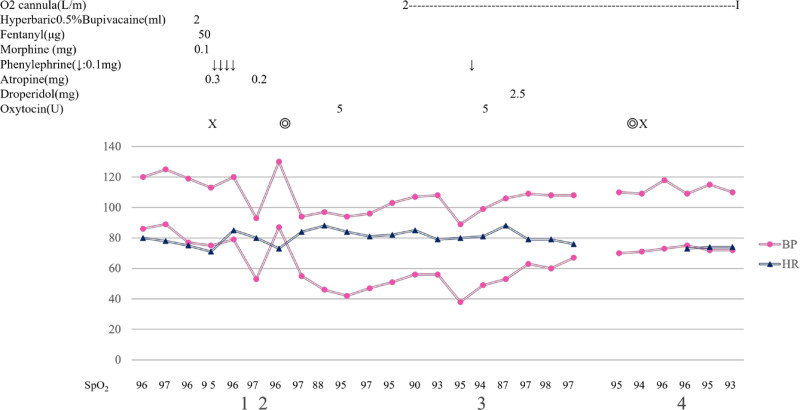
(1) The anesthetic height was ≤ Th4. (2) The infant and placenta were delivered. (3) The patient was nauseated. (4) The anesthetic height was ≤ Th7. Th = thoracic vertebra.

A 28-year-old primigravida with a history of insulinoma resection was transferred to our hospital at 36 weeks and 1 day of gestation. She gained a body weight of 7.5 kg during the 2 weeks before transfer, with remarkable edema in the extremities. Blood pressure was 118/77 mm Hg, pulse rate was 75 beats/min, and percutaneous oxygen saturation (SpO_2_) was 98%. Laboratory data were remarkable for Hb 7.7 g/dL, Hct 27.8%, TP 4.5 g/dL, AST 36 U/L, ALT 29 U/L, albumin 2.4 g/dL and d-dimer 3.4 g/mL but urine protein was negative, and ultrasonography revealed no deep vein thrombosis. Ultrasound examination revealed polyhydramnios and fetal hydrops. The following data were obtained: estimated fetal weight 2909 g (+1.3 standard deviation), biparietal diameter 99.3 mm (+3.3 standard deviation), head circumference 34.96 cm, amniotic fluid index 45.2 cm, and amniotic fluid pocket 12 cm (Fig. 1A). The patient initially suspected to have deep vein thrombosis or preeclampsia, but tests ruled this out. Although the cause(s) of maternal edema and fetal hydrops could not be ascertained, continuation of the pregnancy was considered a risk, and an emergency cesarean section under spinal anesthesia was conducted when the patient was 36 weeks and 2 days pregnant (Fig. 2). There was a decrease in antithrombin III (59%), and 3000 U of concentrated human antithrombin III (Neuart^®^) were administered intravenously before cesarean section. To establish spinal anesthesia, a puncture was made through the 3rd and 4th lumbar intervertebral spaces using a median approach, and a combined solution of hyperbaric bupivacaine 10 mg, fentanyl 25 µg, and morphine 0.1 mg was administered. The sensory block reached the third thoracic vertebral level 3. The duration of surgery and anesthesia was 68 and 83 minutes, respectively. Blood loss was 970 g, and the amniotic fluid volume was approximately 5 L. The newborn’s Apgar scores were 2 and 5 at 1 and 5 minutes after delivery, respectively. The birth weight was 2410 g.

During emergency cesarean section under spinal anesthesia, the patient complained of nausea and was treated with droperidol 2.5 mg. The SpO_2_ suddenly decreased to 86% (room air). Oxygen (2 L/min) was supplied via a nasal cannula and the C-section was completed. The patient returned to the ward after confirming that the level of anesthesia had decreased. Although dyspnea was absent, hypoxemia persisted for 2 days after delivery (Table 1), and computed tomography demonstrated bilateral pleural effusion and mild lung edema (Fig. 1B). Oxygen supply was restarted until the pleural effusions and lung edema spontaneously resolved. The patient’s leg edema also improved after the C-section: the bilateral thigh and lower leg circumferences were ~43 and ~31 cm on the 6th post-cesarean day, respectively, reduced by − 10 cm from the time of hospitalization. The patient’s weight on the 7th post-cesarean day was 52.7 kg, with a fall of − 5.2 kg from the 2nd post-cesarean day. The patient was discharged with good progress on 13th post-cesarean day.

**Table 1:** Results of the patient’s postdelivery arterial blood gas analysis.

	PCD 0	PCD 1	PCD 2
F_I_O_2_	0.32	0.21	0.21
pH	7.341	7.447	7.398
PaCO_2_	42.9	35.4	42.7
PaO_2_	85.9	62.2	62.1
HCO_3_	NA	24.1	25.7
BE	−2.3	0.6	1.3

The infant was edematous and had heart failure with ventriculomegaly. The trachea was intubated immediately after birth and was managed by a neonatologist. The infant was later diagnosed with congenital myotonic dystrophy, and it was discovered that there was a family history of myotonic dystrophy in the mother’s family.

## 3. Discussion

We encountered a patient with mirror syndrome who experienced hypoxemia during an emergency cesarean section due to sudden maternal pulmonary edema that gradually resolved after delivery. Mirror syndrome is associated with high fetal and maternal mortality rates. Chen et al reported that the incidence of mirror syndrome among 98,484 deliveries was 0.0162%, whereas that among patients with fetal hydrops was 23.2%.^[5]^ They also noted that compared to the patients in the non-mirror syndrome group, the uric acid, lactate dehydrogenase, creatinine, and d-dimer levels were significantly, higher and the hemoglobin, hematocrit, platelet, and serum albumin values were lower in the mirror syndrome group.^[5]^ In the present patient, the maternal symptoms along with fetal hydrops were edema, anemia, hypoalbuminemia, and a higher d-dimer level. In addition to fetal hydrops and maternal edema, these laboratory anomalies may help clinicians diagnose mirror syndrome during the perinatal period.

Pulmonary complications, including pleural effusion, hypoxemia and pulmonary edema also had to be considered whether be associated with mirror syndrome. Our patient’s hypoxemia during anesthesia was considered anesthetic-induced somnolence but was diagnosed during the postpartum period as being due to atelectasis and pulmonary edema. Her hypoxemia could have been made more severe by the decision to continue the pregnancy. In a systematic review of fetal associated conditions, maternal edema occurred in 80% to 100% of cases, hypertension occurred in 57% to 78%, and pulmonary edema occurred in 21.4%.^[2]^ Pulmonary symptoms persisted after delivery, as maternal symptoms were reported to resolve 4.8 to 13.5 days after delivery.^[2]^

Pregnant women with suspected mirror syndrome require attention not only for the possibility of worsening respiratory symptoms during a C-section, but also for respiratory symptoms after the C-section, and some patients may require close observation in the intensive care unit. In this case, we could not diagnose mirror syndrome, so the patient was sent back to the ward after C-section. However, if we had suspected mirror syndrome, we could have chosen to treat the patient in the intensive care unit and provided better care. Mirror syndrome has been reported by obstetricians and pediatricians; Nonetheless, reports by anesthesiologists are limited. Not only obstetricians and pediatricians, but anesthesiologists also thorough knowledge of mirror syndrome will contribute to the best anesthetic and post-cesarean section management for patients and reduce maternal and infant mortality.

As the fetal prognosis in mirror syndrome is very poor (live birth rate is 7.7%), the decision to perform an emergency cesarean section can avert fatal and maternal deaths.^[1]^ The present patient’s newborn was diagnosed with congenital myotonic dystrophy, which is known to be associated with nonimmune fetal hydrops (NIFH). NIFH accounts for almost 90% of all fetal hydrops.^[6,7]^ The common pathophysiology underlying many etiologies of fetal hydrops is an imbalance in the regulation of fluid transfer between the vascular and interstitial spaces, with increased interstitial fluid production or decreased lymphatic return. When capillary filtration is higher than the lymphatic fluid removal capacity, the fetal interstitial space is enlarged, leading to fetal hydrops.^[8]^ The precise pathogenesis of NIFH depends on the underlying disorder and remains unclear in several cases. The most common etiology of NIFH is cardiovascular (17%–35%), followed by chromosomal (7%–16%). It has been estimated that the cause of hydrops can be determined in approximately 60% to 85% of cases, although this estimation included postnatal evaluations.^[5]^ It was also reported that the treatment of NIFH can improve mirror syndrome, and many patients (including those without a treatable etiology of NIFH) require immediate delivery because of concerns about worsening maternal disease due to the treatment terms of NIFH.^[9]^

In conclusion, we encountered a young adult woman with mirror syndrome who developed hypoxemia during emergency cesarean section. Pulmonary complications associated with mirror syndrome should be noted during the perinatal period.

## Author contributions

**Conceptualization:** Shino Ichikawa, Satoko Noguchi.

**Investigation:** Shino Ichikawa, Kurumi Saito.

**Writing – original draft:** Shino Ichikawa, Junichi Saito.

**Writing – review & editing:** Kazuyoshi Hirota.
